# Practicalities of implementing burden of disease research in Africa: lessons from a population survey component of our multi-partner FOCAL research project

**DOI:** 10.1186/s12982-022-00113-y

**Published:** 2022-06-07

**Authors:** Binyam N. Desta, Tesfaye Gobena, Custodia Macuamule, Olanrewaju E. Fayemi, Christianah I. Ayolabi, Blandina T. Mmbaga, Kate M. Thomas, Warren Dodd, Sara M. Pires, Shannon E. Majowicz, Tine Hald

**Affiliations:** 1 School of Public Health Sciences, University of Waterloo, 200 University Ave. West, Waterloo, Ontario N2L 3G1 Canada; 2 College of Health and Medical Science, Haramaya University, Haramaya, Ethiopia; 3 Faculty of Veterinary, Eduardo Mondlane University, Maputo, Mozambique; 4 Centre for Research, Innovation, and Collaboration/Department of Biological Sciences, Mountain Top University, Prayer City, Nigeria; 5 Department of Microbiology, University of Lagos, Lagos, Nigeria; 6 Kilimanjaro Clinical Research Institute, Kilimanjaro Christian Medical Centre, Moshi, Tanzania; 7 Kilimanjaro Christian Medical University College, Moshi, Tanzania; 8 Centre for International Health, Dunedin School of Medicine, University of Otago, Dunedin, New Zealand; 9 Risk-Benefit Research Group, Technical University of Denmark, Lyngby, Denmark; 10 Research Group for Genomic Epidemiology, Technical University of Denmark, Lyngby, Denmark

**Keywords:** Partnership, Population survey, Collaboration, Experiences, Africa, Foodborne disease burden

## Abstract

**Background:**

Collaborative research is being increasingly implemented in Africa to study health-related issues, for example, the lack of evidence on disease burden, in particular for the presumptive high load of foodborne diseases. The FOCAL (Foodborne disease epidemiology, surveillance, and control in African LMIC) Project is a multi-partner study that includes a population survey to estimate the foodborne disease burden in four African low- and middle-income countries (LMICs). Our multi-partner study team had members from seven countries, all of whom contributed to the project from the grant application stage**,** and who play(ed) specific roles in designing and implementing the population survey.

**Main text:**

In this paper, we applied Larkan et al.’s framework for successful research partnerships in global health to self-evaluate our project’s collaboration, management, and implementation process. Our partnership formation considered the interplay and balance between operations and relations. Using Larkan et al.’s seven core concepts (i.e., focus, values, equity, benefit, communication, leadership, and resolution), we reviewed the process stated above in an African context.

**Conclusion:**

Through our current partnership and research implementing a population survey to study disease burden in four African LMICs, we observed that successful partnerships need to consider these core concepts explicitly, apply the essential leadership attributes, perform assessment of external contexts before designing the research, and expect differences in work culture. While some of these experiences are common to research projects in general, the other best practices and challenges we discussed can help inform future foodborne disease burden work in Africa.

## Introduction

### Background

The need for multi-partner action research is growing worldwide [1, 2], and collaborative research is being increasingly implemented in Africa to study various health issues [3]. In Africa, a collaborative approach is inevitable to address the lack of evidence on disease burden, in particular for the presumptive high load of foodborne diseases [4–7]. Thus, we implemented FOCAL (Foodborne disease epidemiology, surveillance, and control in African LMIC), a multi-partner study to estimate the foodborne disease burden in four African low- and middle-income countries (LMICs): Ethiopia, Mozambique, Nigeria, and Tanzania. FOCAL is an international collaboration of multiple institutions [8], and includes a population survey, which aims to estimate the incidence and distribution of diarrhea in the community in rural and urban settings.

Although collaborative research is desirable, the knowledge and skillset demanded to successfully implement multi-organization studies are still evolving [1, 9]. The success of a multi-partner research undertaking mainly relies on the quality of the collaboration [10]. In this paper, we applied Larkan et al.’s framework and seven core concepts (i.e. focus, values, equity, benefit, communication, leadership and resolution) for successful research partnerships in global health [11], to self-evaluate our project collaboration, management, and implementation process, focusing on the population survey, thereby providing insights into the practicability of the burden of disease studies in African settings. Here, we present an overview of FOCAL, describe our population survey working-group, and present the results of our self-evaluation, as well as discuss additional best practices and challenges we encountered that did not fit within the framework.

### Overview of the FOCAL project

Starting with the grant call, the FOCAL project leads from Denmark took the initiative to craft the project’s outline, and invited interested collaborators from the four study countries in Africa and supporting partners. Working together, this larger FOCAL team collaborated to design a proposal aimed at facilitating implementation of integrated public health and food-safety surveillance in African LMICs, a priority goal since only a few countries in Africa prioritize implementing surveillance and mitigation strategies to combat foodborne diseases [4, 12]. The whole FOCAL team contributed to the project starting from the grant application stage, and our multiple regular meetings enabled the active engagement of all partners at all times. Upon proposal submission, we allocated and finalized the funds required for the research undertakings in each country, including the source of the various budget items. We secured the funding and started the project on November 26, 2018.

FOCAL spans four years, and annual progress reports precede the yearly-allotted release of funds to partner institutions. Any failure by partner institutions to report the achievement(s) per the specified task(s) in time requires justification, to secure the funding for the subsequent year. Among the project's official documents is a work plan outlining specific activities, milestones, and deliverables, with timelines and responsible partner(s). The project’s proposal-narrative document incorporated risk mitigation strategies that specified the penalty/consequence of unnecessary delays or lack of contribution to the outlined roles. The Principal Investigator (PI), i.e., the contact person for the funders, monitors activities and achievements, and makes executive decisions when needed. Beyond the original partners named in the grant proposal, postgraduate students and postdoctoral fellows have been enrolled via formal procedures of the respective partner institution, with the PI reviewing candidates’ qualifications to ensure transparency. Partners supervised their trainees’ ventures within their respective institution’s requirements and timelines vis-a-vis the project’s aim. Overall (with the exception of disruptions introduced by the COVID-19 pandemic circa March 2020), there were no substantial flaws in the project activities.

For the population survey, we received clearance from nine ethics committees in six countries, put in place three bilateral data sharing agreements, completed the preparation phase, and commenced survey data collection on February 17, 2020.

### The FOCAL population survey working group

The population survey, like the other component FOCAL studies [8], has a lead and a working group, and the partners from Canada are taking overall responsibility for the population survey component. Designated team members from the four African countries who are part of this working group work closely with the partners from Canada and manage the in country-specific survey activities.

The working group comprises members from Ethiopia, Mozambique, Nigeria, Tanzania, Denmark, Canada, and New Zealand. Partners leading the four country-specific surveys in Ethiopia, Mozambique, Nigeria, and Tanzania are located and affiliated with institutions in the countries. Except for the partner from New Zealand, who resided in Tanzania, the partners from Denmark and one of the two from Canada are also located and affiliated with institutions from these countries. The second partner from Canada is originally from Ethiopia, which was an added advantage to this collaborative work. The partners from Denmark and Canada are experts in the field, who are involved in foodborne disease burden studies in other countries, and also part of the World Health Organization’s (WHO) work on the global burden estimates of foodborne diseases, which brought the necessary expertise and experiences to the team.

In general, FOCAL working groups are mostly non-exclusive, where one partner can be a member of several working groups. This helped the population survey working group coordinate with the other working groups to undertake FOCAL’s commitments. For this paper, however, we focus on the collaboration within the population survey’s working group.

### Rationale and application of larkan et al.’s conceptual framework

Larkan et al. developed a conceptual framework aimed to inform partnerships in global health research [11]. The framework highlights the significance of relational and operational aspects of collaborations and presents viewpoints on seven equally relevant core concepts: focus, values, equity, benefit, communication, leadership and resolution. The framework also outlines the process that most global health research partnerships follow: formation, implementation, monitoring and evaluation. Larkan et al. suggest their framework can inform new collaborations or improve existing ones (like ours), but acknowledge that the framework still needs validation.

We picked Larkan et al.’s framework to self-evaluate our collaborative process, because we found their under-pinning evidence credible and suitable to our collaboration. Specifically, we valued that the framework’s development incorporated experience from all LMICs and supporting partners who took part. Additionally, our partnership process suited Larkan et al.’s recommendation for well-functioning collaborations (i.e., agreement on the shared minimum programme, involvement of partners from the design stage, and specific allocation of resources). Finally, we found comparable evidence in other, more recent literature to support the framework’s components, which increased our confidence in applying it. We conducted our self-evaluation while in the midst of survey implementation, to identify areas for our own improvement for the remainder of our collaboration. Each of the partners was asked to answer the same list of open-ended questions, prepared based on the framework components, with items asking about best practices and challenges that did not fit within the framework, and answers were collated.

Two aspects of the framework made it easy to use in our self-evaluation. First was the elaborated concepts and attributes for desirable partnership qualities, from which their core concepts emerged. Second was the explicit recognition for flexibility in application, to allow contextualization of political, social, and cultural realities. For example, when referring to culture, Larkan et al. emphasized partners’ organizational contexts, and we expanded this to also consider work culture related to the local inter-personal and social contexts of our diverse locations.

## Main text

### Formation of the partnership: operations and relationships

When our team of researchers from seven countries came together for the 1st time in the FOCAL project, the driving force that helped us move forward in the collaboration establishment process was a mix of operations and relationships. By operations, we mean that we established our collaboration on a set of activities, which each partner contributed to, with a common goal and shared benefit. Corresponding to Nyström et al.’s observation, the operational aspect of our partnership was largely in place at the formation stage [2]. Here, the formation phase entailed the process starting from the simple communications to initiate the collaboration, to the signing of the sub-grantee agreement between the lead and partner institutions to commence the project activities. Our focus on the project outputs at the formation stage considerably helped us in accommodating the varying work and communication cultures of multiple partners.

Relationships were also key facilitators of the partnership formation process. Partners came together for FOCAL in part via pre-existing connections, a concept described by Duff [13]. For instance, some of our partners worked together on other collaborative projects, which significantly reduced hurdles (e.g., in terms of time, interactions, and trust) and helped nurture additional relationship formation between collaborators. The relationships among partners strengthened as the partners continued working on the early operational features of our collaboration (described above), which aligns with Boucher et al.’s concept of the setting of a shared aim and interest [14]. The interplay and balance between operations and relationships early in our partnership formation enabled smooth survey design, and helped to facilitate survey implementation.

### Implementation phase: the seven core concepts

Below is our assessment of the strengths and gaps in survey design and implementation, by Larkan et al.’s seven core concepts. Overall, we felt the gaps presented below, while important to identify, have not been substantial flaws nor affected FOCAL’s deliverables and timeline.

### Focus

When designing the population survey (as well as the overall FOCAL project), and as suggested by Leone Sciabolazza et al.[15], we set shared goals and aims of estimating the burden of the foodborne disease in the respective African countries. We perceived that all partners have a common understanding of the population survey's objective, aided by the fact that we are all are researchers in the field who are well aware of the existing knowledge gap concerning the burden of foodborne diseases. Of the project’s primary outcomes, a list of the specific survey aims was shared with the working group members by the leads for review. This was revised iteratively by the working group collectively, until each member acknowledged the credibility and achievability of the objectives. The partners also expressed their enthusiasm and determination to produce practical knowledge to share with the scientific community, their respective countries, and Africa broadly. The focus on our shared goals was evident from the emphasis we gave to the process of survey designing, our keenness in providing inputs to developing the survey protocol and tools, and our eagerness and focus on achieving milestones. Moreover, the motivation to enable smooth survey implementation by resource mobilization, the enthusiasm to attend regular meetings, our proactive plan to engage stakeholders in the process, and meeting the survey’s timeline were among the examples showing our focus on the shared goals.

In a few instances, we observed a lack of timely execution of specific tasks, even if we had previously reached an agreed timeline at a team level. In general, we acknowledge the need to improve this, but we are mainly focused on building on our successful practices (e.g., shared goal of generating data on the burden of foodborne diseases in African contexts; shared experiences as foodborne disease researchers).

### Values

While operating together, we recognized slight variations in work culture, which, as noted by Gélinas [10], is to be expected as we came from different institutions in multiple countries. Situations where these variations were noticeable included inconsistencies in sharing accountability on overdue or undone tasks, timeliness on notifying changes or challenges, and voluntarily taking leads on extra responsibilities and delegation of duties. In some instances, we also noted performance variation among our country-specific study teams in the field, which improved when the country collaborators/ supervisors are physically present. We preferred not to look at these disparities as challenges or limitations, rather we considered them as an opportunity to share experiences. Fortunately, the open-mindedness of all partners to change(s) enabled us to entertain each variation accordingly.

Our partnership, related to Stanley and Anderson’s recommendation [9], is built upon trust, which we believe is key to the successful designing and implementation of our survey (beyond the official communications, memoranda of understanding or agreements signed between partner institutions). Nevertheless, we also encountered gaps in sharing or delegating responsibilities within the team. We are sometimes inclined to overburden ourselves with multiple activities even when there are official delegates for the respective tasks, thereby delaying the timely completion of assignments. We have identified that we need to be better at sharing responsibilities to maintain our effort in meeting the project’s timeline (particularly given the COVID-19, which creates various disruptions that differ by location and over time).

We feel that our level of commitment to perform the tasks and achieve the milestones of executing the population survey is high, and we attribute it in part to our motivation for scientific contribution, our desire to continually strengthen our professional inter-relationships, and the leadership role of the PI. We all experienced missing meetings without giving advanced notice, and there were also instances where group-level presentations took place without having complete information, due to our untimely response and unavailability. However, we felt that any negative impacts of these variations were minimized in part by having multiple people per institution (thus allowing a ‘back up’ to be contacted).

### Equity

We are inclined to assume that our partnership is inclusive, as a recommended practice for collaborative researches [3, 9], because we have been jointly involved in almost every decision made from the formation stage, and each partner appropriately contributed to the work. We also considered students involved in the survey as equitably contributing to decisions, referring to their participation in every meeting and activity as applicable. In some instances, we also restricted our group-level decisions to pre-determined alternatives based on available evidence, or expertise (e.g., survey design, local acceptability). However, we have not further ascertained whether group-level decisions are reflective of the inclusiveness of each partner’s interest. For example, some of us may not have shared thoughts in instances where keen participants urged the group towards a particular decision point. Moving forward, we recognize the need to look into more ways of making sure our decisions are undoubtedly inclusive.

We established the various FOCAL working groups by considering expertise and interest and allowed group members to self-identify (i.e., we left no team members out of the working groups in which they wanted to participate). For example, the overall lead of the population survey was an expert with extensive experience of doing similar population surveys in other countries. We entertained all inputs from each partner with respect and acknowledgment of their expertise at all levels of survey design and implementation, as advised by Stanley and Anderson [9].

When planning the survey data collection and developing the survey tool, we interacted with respect and recognition, and we contracted (i.e., at the project level) a gender specialist to inform the survey and ensure its sensitivity to gender differences across the project cycle. In crafting the field survey, the leads put forward a draft process, which each partner reviewed, and collectively we discussed content and finalized the design. The survey tool was developed in the same way: the leads put forward a draft survey tool, and all partners reviewed each item and provided inputs mainly to contextualize and address country-specific issues. Additionally, each partner took the lead to facilitate translating the survey tool into the respective local languages (Ethiopian–Amharic and Afaan Oromo, Mozambique–Portuguese, Nigeria–Yoruba, Tanzania–Kiswahili), thereby enabling the smooth survey undertaking. We took this process as evidence that our collaboration relied on and recognized the contribution of each partner.

In an effort to balance potential power differentials that might emerge between the members from the study countries and survey leads (given the credentials of the overall survey lead), different approaches were in place. These approaches included explicitly delineating the stakes of each partner and flexibility in some of our premade decisions. We did not expect any power imbalance, as demonstrated by Essabbar et al [16], between the study countries, where independent surveys were administered. Having each partner country take a turn to host the FOCAL annual meeting also contributes to power equitability in our collaboration. Despite this, we acknowledged some could experience what Duijs et al. expressed as a sense of disregard [17], considering instances where inputs/comments from partners got refuted with reasoning. Given the partners’ level of expertise and experience, refuting inputs could have slightly impacted the sense of ownership, thereby influencing the eagerness and devotion towards achieving our goals. We, as a group of researchers, recognized that we assess every thought put forth in our discussions from our different areas of expertise and experience. Moving forward, we agreed to continue doing this appraisal, as not all ideas can be feasibly implemented.

As described earlier, during the grant writing process, we estimated the required budget for country-specific surveys and made decisions beforehand, which guaranteed the fair sharing of resources among institutions/countries. However, as we finalized the population survey design, we realized we needed to change from web-based only data collection, to both face-to-face and online data collection. To allocate resources to accomplish this, we re-assigned resources from one country’s budget to the other, and also reallocated items within a country budget. Thus, the resource sharing took place in consultation and agreement with every partner, based on pre-set activities and roles, to enable an adequate and fair share of resources. Moving forward, we noted the need to scrutinize resource sharing and reallocation due to the COVID-19 pandemic.

### Benefit

Starting from the partnership formation stage, as proposed by papers on collaborative research [3, 13], we strived to encourage mutual benefits among partners, which resulted from taking part in both the population survey research, and the broader collaboration itself. As partners in a large-scale study in Africa, the experience will be a rewarding one, both to the collaborators and their institutions. The partners from Africa may benefit greatly from this study as it could provide information to enhance control strategies for foodborne diseases (e.g., set a platform to ensure food safety and surveillance of foodborne diseases in LMICs). In this regard, engagement of stakeholders from each study country was planned throughout the project, which included their invitation to our FOCAL annual meetings. Our intent with this is to create a sense of ownership and enable the study outcomes' utilization, thereby letting stakeholders realize the study's contribution to their country. We believe the need to keep engaging the stakeholders inclusively to ensure that our research delivers the intended purpose (e.g., set an advisory group), following an integrated knowledge translation approach [18].

In terms of scientific contributions, the working group members will all be authors on publications coming out of the population survey (unless they decline or choose acknowledgement), and other FOCAL team members will be acknowledged accordingly. This authorship strategy (which also includes prominent inclusion of trainee co-authors) was discussed and agreed on in various team meetings. Moreover, students from member countries have actively participated in the survey design and implementation, thereby allowing them to develop the research skill to help combat foodborne diseases at various levels. Students will also be lead authors of publications coming out of the survey when they play a leading role on the part that would form their thesis. We acknowledged the need that students involved in country-specific study teams, who play significant roles in our field data collection, be aware of the mutual benefits. We learned the necessity to explicitly assert that we share benefits mutually at all levels of our study-teams.

### Communication

Corresponding to the recommended practice in partnerships, we strived to build our partnership with open and transparent communications among partners [10, 19]. We used email, and group voice and video calls for every interaction, and we plan in-person gatherings once in every project year. We also have a common password-protected share web-site at the PI’s institution, where all the necessary documents of the project are archived and communally available. We have fortnightly meetings, with circulated agendas beforehand and distributed meeting notes immediately after, on which every partner is free to comment on or provide inputs. We also include explicit action points in meeting notes (with responsible individuals identified) to guide us on the urgent/immediate tasks. At these regular meetings, the lead and each partner give updates on the survey progress and any related notifications. So far, partners owned the tasks and took accountability, which helped to evade any power hierarchy, thereby enhancing the openness in our communications. We discussed or informed each other of any issues of the survey components, and also openly discussed and agreed on benefits coming out of it. These efforts, in turn, enhanced our communication in terms of honesty and unambiguity.

On top of regular meetings, partners communicate via email to provide updates, discuss any issues or make enquiries, request support, share documents, follow-up on tasks, and schedule meetings. There is no specific communication chain to follow to make a connection or interact with others on the team; rather, communications are linked by involving project leads and interested team members (by copying in emails), which allows for transparency within our conversations. Our email communications use layman wordings and positive language, and include warm greetings, best wishes, and sometimes sharing of not-too-personal details (e.g., achievements, major life milestones), the latter of which have emerged more recently as a result of the experience sharing. Also, following almost every email was timely feedback with gratitude. Separate voice/video calls were also our alternative communications to facilitate the survey as deemed necessary. Our first in-person meeting to launch the project (held in Addis Ababa, Ethiopia, in February 2019) enabled ease of interaction between ourselves. We also used online training sessions to help bringing everyone on the same page regarding data collection tools and procedures. We believed these collective efforts allowed us to foster further openness and honesty in our communication.

On the other hand, there were instances where we failed to: give feedback urging timely actions/changes; execute decision/action points in a timely manner; attend one-on-one meetings; communicate survey progresses, updates, challenges, or general comments in a timely manner; and actively contribute to group conversations. We recognized that these gaps are affecting our communication efforts, and moving forward, we are committed to improving each of these gaps.

### Leadership and resolution

In our partnership, we intended to value leadership and resolution attributes suggested for collaborative action research [2, 3]. Upon the project's inception, the PI considered credentials when designated the survey lead, in particular, recognized the expertise evident from doing similar surveys in other countries. We outlined the roles and responsibilities of each working group member in the survey protocol. Timeframe and milestones were in place to enable partners to monitor their performances and meet the project's timeline. Partners from each study country managed its budget to accomplish the respective tasks. Signed memoranda of understanding and data transfer agreements, the study protocol, and meeting notes were among formal documents that delineate the duties and accountabilities of each partner. Partners were solely in charge of delivering the respective country-specific milestones, which in turn aided the study process.

Specifically, every partner was mindful of the accountability entailed in accomplishing the tasks. The survey leads guided the activities and oversaw the progress on designated tasks. We tried to apply a delicate leading, probing, or following up roles, which gave due regard to sensitive issues, tactical approaches, and balancing. Overall, the project’s lead monitored the activities and played a vital role in balancing the operational and relationship features among partners. As we remarked earlier, hierarchical positionality or power imbalance have not been noticeable in our partnership, which also linked with the full and equitable delegation of tasks. According to Morrison-Smith and Ruiz [20], given the challenge with quality interaction in geographically dispersed study teams, hierarchical leadership is not be the best approach, and empowering the team members is key. We had risk mitigation plans and strategies to deal with or cope with difficult or challenging situations concerning the survey implementation. As noted above, we set and agreed upon a binding penalty that every partner could face for being unable to comply with or meet the requirement of operating within the timeframe. Even if very unlikely to happen, the penalty extended to the reallocation of funds to other partners/activities.

We continued to deliver the tasks with determination and perseverance, for example, by working out of office hours (to accommodate time zone differences) and on the weekends, voluntarily taking on extra duties, and completing some assignments in advance. We dealt with challenges by being flexible with some premade decisions (e.g., reassigning budget items to distinct tasks) and by investigating alternative ways of executing the action points. The attributes of leadership and strategies of resolution we applied depreciate conflict among partners.

Regardless, we noticed slightly varying performances among ourselves in terms of time and efficiency. For instance, we noted irregularities in the time vs. completeness/contextual appropriateness of survey translations. Also, we encountered variations in the starting date of data collection vs. data quality. The disparities had varying effects and many implications, which we could resolve with attributes linked with management or resolution. These attributes could include activities such as: give due attention to detail in all activities; step-by-step follow up and address concerns; divide tasks into smaller pieces and provide encouragement and recognition for each accomplishment; strengthen technical support as applicable; and create platforms to validate achievements [20]. In general, we perceived that sounder achievements entail multiple aspects, including comprehensive leadership and resolution roles, to which we aspired and strived to fulfil.

### Outcomes: increased capacity, influenced practice and policy

When considering our surveying process, and Larkan et al.'s core concepts, we foresaw short and long-term research outputs akin to other collaborative efforts [10]. The short-term outcome of increased capacity included recruitment of postgraduate students and postdoctoral fellows from partner countries. These trainees are actively engaged in the population survey and work jointly with partners, which eventually will equip them with practical research experience and skills [21]. The experiences and skills could be in designing stand-alone surveys, working within a group of various collaborators, seeing the ups and downs in field surveys, acquiring technical know-how of survey instruments and tools, and developing analysis, reporting, and writing skills. Moreover, they will obtain skills in communication/interaction, evaluation, multidisciplinary thinking, socialization, leadership and resolution. In addition to capacity building, we anticipate that our collaborative effort will contribute to both African and global disease burden reduction efforts, as it will provide evidence to inform policymaking and other changes. We believe that we regularly need to take steps to reinforce our partnership, since we feel that keenness and dedication from every project member and stakeholder in a collective sphere is necessary to achieve the desired outcomes.

### Other best practices and challenges

Here, we share other successes, that we put forward as potential best practices, as well as challenges we experienced in our collaboration, that did not directly link with partnership issues explored above.

### Assessing external contexts

At the beginning of the project, we discussed feasibility issues and the uses of planned outputs within our local study teams and with local health, agricultural, and livestock sector representatives in each study country. The local discussion agenda included project objectives, activities, and deliverables, the usefulness and applicability of the study outputs, and experiences with previous studies (if any), including best practices and challenges. These discussions informed our study by helping to evaluate the feasibility or achievability of the project aims given population characteristics, infrastructure, and end knowledge use. For instance, we added an in-person data collection method to our plan of the web survey, because of population characteristics (i.e., literacy status) and infrastructure (i.e., access to technology). The local stakeholders confirmed the utility of anticipated study results for their mandates.

Through these discussions, some stakeholder institutions initially asked to be the local host partner and have a direct role in coordinating the project activities. This desire arose from the benefits (in money and kind) they had previously obtained from coordinating other projects in the health field. Because of this expectation and because our academic team members were already the local host partners, some stakeholders were initially discontent and, as a result, had little interest in providing support. To alleviate this, we held formal and informal discussions with the leads/representatives of these institutions to explain the structure of our project administration and the responsibility and funding details for each task in each institution. Through these meetings, we built relationships with some of these stakeholders.

In addition, we hosted a discussion forum in Ethiopia, with invitees from all the above-listed sectors in all four study countries, and international stakeholders (e.g., from other ongoing similar projects, funders, and non-governmental organizations residing in Ethiopia). The forum started with an overall discussion with all stakeholders from all four countries. The discussion covered issues related to food safety and foodborne diseases in the countries and globally, challenges to ensuring food safety, and how surveillance of foodborne diseases can be done in collaboration with different sectors. Then, stakeholders discussed the above-listed issues in more detail, in their country-specific groups. During these within-country discussions, additional stakeholders were identified, including those with overlapping roles and responsibilities for food safety, including: health; agriculture and rural development; environment; industry, trades and investment; science and technology; and food science and technology. We then approached these additional stakeholders to explain the study and its planned outputs, and they confirmed they wanted to receive the study outputs and apply them within their efforts.

### Involvement of community/religious leaders

Active cooperation with local community/religious leaders played a pivotal role in survey implementation. For example, in Nigeria, the village heads and community leaders—known as *Baalẹ—*worked closely with community residents and engaged them in our study. In Mozambique and Tanzania, local authorities (community leaders) were more influential in engaging the communities than religious leaders. In Ethiopia, local leaders, and personnel from sectors such as health, agriculture, and livestock, were more involved than religious leaders. Before starting the survey, we invited these local/religious leaders in each study site to meet with the Health Extension Workers (HEWs)/Community Health Workers (CHWs) and data collectors. The responsiveness and approval of the local leaders resulted in getting adequate information on context- and location-specific issues (e.g., local norms and values). This information helped lessen the potential for subjecting study participants to emotional and cognitive difficulties(as described by Ting [22]) via respecting the local norms and values and carefully handling sensitive issues. For instance, some women in some sites were shy/discomforted being interviewed by a male data collector particularly being asked about their experience with diarrhea. We addressed this by having a gender-inclusive data collection team, and our female data collectors conducted such interviews. Moreover, the questioning about diarrhea was preceded by statements such as 'we all experienced diarrhea at least once in our life’ using the local language.

In some countries, local/religious leaders also facilitated survey administration by grouping the target villages into clusters based on their geographic locations and assigning specific dates for data collection in each locality. In some instances, the village heads also volunteered to promote the survey in their community before the agreed day for data collection. This involvement by village heads and community leaders improved access to the targeted population groups and improved the response rate. In a few encounters, when some community members were suspicious and hesitated to participate in the survey, the village heads and community leaders helped explain the benefit of the project to the community to promote survey participation.

However, we were unable to reach out and mobilize the whole segment of the community in some sites with the local leaders we contacted. To alleviate this, a local study team member regrouped with the community leaders to identify the missed segments of the study sites, which, in turn, helped us in logistical preparations for data collection (e.g., number of data collectors and days needed). At first, we underestimated the number of community leaders needed to access households in some sites, which we reconsidered and contacted more community leaders afterwards.

In some countries, we also held an on-site training for our study team, and the local leaders attended the training and explained the local values, norms, and beliefs to data collectors. The local leaders were highly engaged in the study starting from before data collection began, and this helped us cope with the interruptions due to COVID-19, specifically, in pausing and restarting data collection as local public health restrictions were implemented and lifted.

### Engagement of health extension workers/community health workers

Mobilization of trained HEWs/CHWs at the various sites facilitated the smooth administration of the survey. Here, as the HEWs/CHWs are known to the community and familiar with the physical layout of the villages/towns, our local study teams were welcomed in most households, and locating selected households was not challenging. Furthermore, because the HEWs/CHWs knew and had contact with the village heads/community leaders in each study site, they were the key facilitators of the successful involvement of these leaders. We engaged the HEWs/CHWs from the beginning, before launching the survey, so that they were able to inform the community about the upcoming research activities. The HEWs/CHWs, demographic- and health-surveillance site field workers in Ethiopia, travelled with the local study team and introduced the local study team members and the study to the approached households. In some countries, training the HEWs/CHWs together with the data collectors helped improve the HEWs/CHWs' level of engagement in the study. This training enhanced the effective and successful administration of the survey by the data collectors at various villages and communities visited because the HEWs/CHWs had a similar level of understanding of the study aims and procedure and were allowed to introduce the study to potential participants, which improved the response rate.

Our data collectors were from our universities, and in some countries, communities preferred when investigators were from health services rather than an in-country university. In one country (Mozambique), the communities cooperated more when researchers wore lab coats (as noted and done by some HEWs/CHWs). This better cooperation might be due to the immediate medical services/treatments the communities have received on previous health campaigns by health care workers—who usually wear lab coats. Although crucial, engagement of the HEWs/CHWs was sometimes challenging as they were involved in many different projects (including related to the COVID-19 pandemic and cholera) and other administrative commitments. In some instances, data collection was delayed due to the unavailability of the HEWs/CHWs. To overcome such encounters, we reworked our enumerators' availability to match with the HEWs/CHWs. We also compensated the HEWs/CHWs in money, which accounted time they spent on project-related activities.

### The use of qualtrics to overcome limited internet access

As noted by others [23, 24], we faced technical challenges in implementing a web-based data collection system in the African setting. In our dual data collection methods (in-person interviews by data collectors and web survey by self-selected participants), the challenges we faced were related to access to internet and technology, and varying literacy status of communities. We used tablets for the in-person interviews in our study sites due to limited internet access. We distributed survey web-links for the web survey administration, but participation relied on access to the internet and technology (e.g., smartphones, computers). For both modes of survey delivery, we used a web-based survey platform, Qualtrics, that allowed us to collect data without the requirement of mobile internet access. Qualtrics offers an offline data collection option through its app available on Google Play Store, enabling offline (without internet) survey completion in the field. Then, the data are later uploaded to the database when the internet is accessible. Additionally, Qualtrics, like other web-based data collection platforms, helps minimize entry errors during data collection by restricting the insertion of implausible values.

### Participants’ expectations for support/incentives

Although most participants were willing to complete the survey without expectation of incentives, some participants expected such remuneration. We identified this issue early during survey piloting, and since we did not have a budget to incentivize study participation, our data collectors addressed this via spending extra time explaining the purpose and benefits of the study to the community. As a result, some additional participants agreed to complete the survey. The COVID-19 pandemic created an opportunity for incentives in one country (Mozambique), where face masks were offered to interviewed participants, which some were satisfied with it. In addition, when data collection resumed after pandemic restrictions lifted, we re-reviewed the purpose of the study with the local leaders as needed.

### Language barriers

In all our study sites, we surveyed using the local languages spoken by most people. In some countries, we needed and translated the study documents to the local language(s). All but one country used one language to survey with, and we translated the survey accordingly. We recruited data collectors who were fluent in the local languages and conversant with the cultural background of target communities. This recruitment facilitated the survey implementation, as our data collectors encountered only some participants who needed clarification or further explanation on survey items or took longer than usual to respond to questions. Though our data collectors spoke each majorly spoken language in the study community, we still encountered people speaking other languages in some countries. To resolve this, we formed local study teams that comprised at least one bilingual member.

Creating a single survey with multiple language options facilitated consistent survey administration and data analysis, and Qualtrics allowed this. Participants or their interviewers chose (and completed) the survey in their language via a drop-down list displayed on the front web page. Programming the Amharic version of the survey into Qualtrics was a challenge, as the character font was not initially compatible with Qualtrics. We resolved this issue by installing the particular enabler software (i.e., GeezIME).

### Obtaining ethical approvals

Our institutional and national requirements meant we obtained ethical clearances from nine review boards (in 6 countries), a cumbersome and time-consuming process that took longer than we anticipated. Ethics committees took between three weeks to more than 6 months to provide their review, in part because some committees had few active members, and because some committee members serve part-time and have other duties and responsibilities, making it difficult for committees to meet and finalize decisions about submitted applications.

We submitted our application to between one and three committees per country; when there were two or three committees, the approval process was sequential (e.g., University review board reviews/approves, then national board reviews/approves), which also extended the time needed to complete the process. The application formats and requirements were different for each country and included materials and data transfer agreements with the project partners. Upon receiving feedback from a respective approval committee, we discussed and entertained country-specific issues raised by the reviewers at the local level and presented the changes with other points that needed cross-country decisions at the project level. We then identified and incorporated the comments that required change to the entire study, from the multi-ethics committees, at the project level, and submitted these revisions back to those boards who required approval over revisions. This process re-occurred multiple times. Translation of the study protocol to the local language by sworn translators was also a requirement in one country (i.e., Mozambique). However, as the sworn translators were not professionals in our field, we foresaw potential alteration of meanings of some contents upon translation. Thus, our local partners, who speak the languages and are experts in the research field, worked closely with the translators and ensured content accuracy.

### Other best practices

The leading role of experts with experience in similar surveys reduced many hurdles in the survey design and implementation, akin to Nyström et al.’s recommendation [2]. We discerned that a smooth working relationship with institutional and other stakeholders, as advised by Munung et al [3], aided the survey process in expediting the research activities, recruiting assistants, students and data collectors, and processing budgets. Akin to Nyström et al.’s proposal, we also noted that engaging graduate students and empowering them, with the oversight of their supervisors, was an efficient approach to achieve the research. Country-specific on-site data collectors’ training, aiming to enlighten them on the uniqueness of each study community, enhanced the data collection process. Moreover, we made our local study teams’ gender-inclusive to address gender-sensitive issues, additionally alleviating potential security threats faced by data collectors when going alone in some study sites.

### Other challenges

We faced several other challenges worth noting. First, there was more than one study running simultaneously in the same locality of some study sites; as a result, some community members were confused and mixed-up questions/responses between the studies. To address this, our data collectors spent more time explaining the distinction. Second, in Mozambique, it was difficult to find males 10–35 years to participate in the study on our 1st and 2nd days of data collection (February 2020). At that time, a rumor (with an unidentified source) was circulating among the public about the compulsory recruitment of young males to join the national army forces, mainly via WhatsApp social media. This could be the reason for not finding young males in the community, because they were avoiding strangers as they do not want to be compelled into the army. Thus, we paused data collection on those days and continued on later dates, and were able to sample from some households’ members lists containing young males. Additionally, as we were going out to the field during working/school hours, we missed some household heads’ or in-school children’s participation in the survey. The inability to get up-to-date household registers and the difficulty to access sampled households (due to the unevenly dispersed houses and poor road-quality) were among the challenges we faced during face-to-face data collection in some sites. To adjust for the extra resource demand resulting from household inaccessibility, we set a few data collection days per month, as our sample size estimation was considerate of resource constraints.

## Conclusion and suggestions

Here, our multi-partner, multi-country team conducted a self-assessment of our collaboration, using an existing framework for partnership, with the goal of improving our own efforts as well as informing future collaborations. We noted that some of the experiences reflected here are common to multi-partner research projects. However, our approach in the self-evaluation allowed a comprehensive and structured presentation of the lessons learned, including issues specific to Africa. We evaluated our partnership formation, which was bolstered by the interplay and balance between aspects of operations and relationships. One feature of our partnership was a focus on shared goals and aims, where we also identified gaps that require improvement. We elucidated the slight working culture differences of our intercontinental collaboration, and identified trust and commitment as our core values, which we evaluated by signifying the need to build more on enhancing responsibility-sharing and dedication. We assessed our partnership to be inclusive, although we recognized the need to be vigilant in handling inputs from partners and ensuring the inclusiveness of our decisions.

Each of us has acknowledged the mutual benefits of taking part in the survey, with the need to ensure that every team member is well-acquainted with mutual benefits. We illustrated the communication features of our interaction (in terms of openness, transparency, honesty, and unambiguity) while showing the existing communication gaps and our intentions to improve on various components. We unanimously agreed that the leadership attributes (and their dependent resolution strategies) described above worked well, and we acknowledge the need to keep with these same attributes and strategies in our remaining activities.

In general, our assessment suggests that successful partnerships need to consider these core concepts explicitly, apply the essential leadership attributes, perform assessment of external contexts before designing the research, and expect differences in work culture. We suggest Larkan et al. use our application of the framework to help validate it, given our mix, experience/expertise, and fit to its components. Moreover, experiences like ours, when presented with the core concepts and other best practices and challenges, can help inform ongoing and other similar surveys, for example, those aiming to study the burden of foodborne or similar diseases in African or other LMICs.

## Data Availability

Not applicable.

## Abbreviations

FOCAL: Foodborne disease epidemiology, surveillance, and control in African LMIC
LMICs: Low-and middle-income countries
PI: Principal investigator
WHO: World Health Organization
HEWs: Health Extension Workers
CHWs: Community Health Workers
